# Improving the Quality of Healthcare Provision Regarding HPV Immunization for Women with CIN2+ Lesions: The Experience of the Veneto Region in Italy

**DOI:** 10.3390/vaccines11040757

**Published:** 2023-03-29

**Authors:** Anna De Polo, Michele Tonon, Filippo Da Re, Sara Rosafio, Elena Narne, Davide Gentili, Silvia Cocchio, Vincenzo Baldo, Francesca Russo, Alessandra Buja

**Affiliations:** 1 Department of Cardiological, Thoracic, Vascular Sciences and Public Health, University of Padua, 35131 Padua, Italy; 2 Directorate of Prevention, Food Safety, Veterinary Public Health, Veneto Region, 30123 Venice, Italy; 3 Screening and Health Impact Assessment Unit, Azienda Zero, 35131 Padua, Italy

**Keywords:** quality improvement, HPV vaccine, cervical screening, preventive healthcare, human papillomavirus, CIN2+

## Abstract

HPV is the most common cause of sexually-transmitted infections the world over. The aim of this study was to assess the impact of a healthcare quality improvement strategy designed to increase the rate of vaccination against HPV in women diagnosed with cervical lesions graded as CIN2 or higher (CIN2+) during routine screening. The Veneto Regional Health Service developed a 22-item questionnaire to measure the gap between ideal procedure and real practice regarding the offer of vaccination against HPV for women undergoing routine cervical screening. The questionnaire was administered to nine expert doctors, one at each of the region’s Local Health Units (LHUs). An additional specific assessment concerned the quality of the related web pages available on the LHU websites. Strategies to close the gap between ideal procedure and real practice were decided collegially, and a checklist to support good practices was developed and shared with operators at the LHUs. Changes in practice were measured using data relating to women diagnosed with CIN2+ lesions extracted from the regional oncological screening database before and after the publication of a Regional Procedure on the topic. The LHUs differed considerably in how they managed each step, in terms of training for healthcare personnel, organization and assessment of the pathway from cervical screening to HPV vaccination, and in dedicated website communication. After implementing the quality improvement strategy, the proportion of women given a first dose of HPV vaccine within 3 months of being diagnosed with CIN2+ lesions at 1st-level screening rose to 50% (compared with 30.85% beforehand), and the median time elapsing between a diagnosis of CIN2+ lesion and a first dose of HPV vaccine dropped from 158 to 90 days. These findings underscore the importance of providing training to promote vaccination for general practitioners and other clinicians. The study also confirms the need for more efforts in communication to ensure that any citizen has the opportunity to access preventive healthcare.

## 1. Introduction

Human papillomavirus (HPV) is the most common cause of sexually-transmitted infection everywhere in the world and basically the sole cause of cervical cancer [1]. According to the latest data from the International Agency for Research on Cancer, the age-standardized cervical cancer mortality rate among Italian women is around 0.78/100,000, with an age-standardized incidence rate that has remained around five new cases per 100,000 women a year in the last decade [2].

HPV malignant cytological lesions are divided into L-SIL (low-grade squamous intraepithelial lesion), including CIN-1 (1st degree cervical intraepithelial neoplasia) and H-SIL (high-grade squamous intraepithelial lesion) including CIN- 2 and -3 (2nd and 3rd degree cervical intraepithelial neoplasia, the latter also including carcinoma in situ). In most cases, the infection causes mild cellular abnormalities that regress spontaneously. On the contrary, the persistence of the virus in epithelial cells is a predictive factor of evolution, over the years, into cervical cancer.

For the treatment of cervical precancerous lesions caused by oncogenic HPVs, surgery (i.e., excisional treatment with local anesthesia, cryosurgery, laser therapy, electrosurgery, or cone biopsy or conization or loop electrosurgical excision procedure) is recommended. Moreover, therapeutic vaccines and genome-editing therapies (such as the CRISPR/Cas platform) targeting HPV E6 and E7 oncoproteins have recently shown promising results in preclinical and clinical trials to control the progression of HPV-related cervical precancerous and cancerous diseases [3].

Vaccination against HPV has proved highly effective (>90%) in preventing infection in women under 25 years old who have never previously been infected with the virus [4]. Cervical screening is another evidence-based strategy for reducing the burden of cervical cancer. In the Veneto region (north-east Italy), a PAP test is offered every three years to 25- to 29-year-old women who were not vaccinated against HPV before the age of 15, while HPV testing every five years is recommended for all women over the age of 30 [5]. Women who test positive are invited to a clinical examination, and if a diagnosis of precancerous lesions or cancer is confirmed, they have conization surgery.

Nowadays, there is global consensus on the recommendation of HPV vaccination for women who undergo conization after being diagnosed with CIN2+ cervical lesions because it has been demonstrated that vaccination is effective in preventing relapses, especially in cases of CIN2-3 [6,7,8,9,10,11].

Despite the evidence in the literature on the efficacy of prevention strategies, the degree to which they are adopted in healthcare practice remains uncertain, and it is worth taking action to promote their implementation. An iterative process consisting of several steps is recommended. This includes: adapting guidelines to local contexts; identifying barriers to their use; selecting and implementing tailored interventions to promote their adoption; monitoring and assessing the associated outcomes, and the sustainability of the recommendations. Quality improvement (QI) in the provision of healthcare is described as a systematic, information-driven, change-focused activity [12]. QI proved vital as a contextual organizational factor in the adoption of evidence-based practices (EBPs) and can be used to support their introduction and consolidation. To significantly influence quality improvements in the provision of healthcare, the application of EBPs needs to be followed up with efforts to overcome the inconsistent incorporation of research findings in clinical practice. More in general, QI in healthcare provision can be seen as a systematic, ongoing effort to solve problems, improve services, and ultimately achieve better patient outcomes [13,14,15].

The aim of this study was to examine the impact of a QI strategy to increase the uptake of HPV vaccination by women diagnosed with cervical lesions graded as CIN2 or higher (CIN2+) during routine cervical screening.

## 2. Materials and Methods

### 2.1. Context

The Italian National Health Service (NHS) is a public system financed mainly by general taxation and organized essentially on a regional basis [16]. Its policies are grounded on fundamental values of universality, free access, freedom of choice, pluralism in the provision, and equity. Healthcare facilities and activities are planned and organized by regional authorities in accordance with a national health plan that is designed to guarantee an equitable provision of comprehensive care throughout the country. The Veneto Regional Health Service (in north-east Italy) is divided into 9 Local Health Units (LHUs), each approximately covering a province. The Service has adopted the current Italian National Prevention Plan [17] and National Vaccination-based Prevention Plan [18], which outline the healthcare procedures and services to be made available to the whole of the country’s population.

In the Veneto Region, immunization against HPV is offered actively and free of charge to all children in their twelfth year of life. It remains available free of charge at a patient’s request for people up to 25 years old and for other at-risk subgroups of the population, such as men who have sex with men, patients with HIV, and women diagnosed with precancerous cervical lesions graded CIN2+ [19].

### 2.2. Quality Improvement Strategy

In November 2020, the Veneto Regional Health Service adopted an evidence-based procedure called “HPV vaccination for women treated for high-grade HPV-related cervical lesions” [20] for the purpose of offering HPV vaccination to the population of women treated for cervical lesions of grade CIN2+ within twelve months after conization.

### 2.3. Identifying the Gap between Ideal Procedure and Real Practice

From May to August 2022, the Veneto Regional Health Service held some focus groups with doctors, healthcare assistants, statisticians, and communication experts to outline the main processes and steps provided by the procedure issued in November 2020 and those necessary to promote its successful implementation, based on the scheme set out in Table 1, in order to define the elements of the survey.

A questionnaire comprising 22 items was then developed (see File S1) and subsequently administered to 9 doctors working at the Vaccinations Unit and thus experts on the topic, one at each of the LHUs in the region, to see how each main process was actually implemented. After they had completed the questionnaire, each expert was briefly interviewed by phone to examine any points in the procedure that seemed to be incomplete, contradictory, or needing clarification.

The answers were scored from 0 to 2, according to their ordered qualitative classification. For example, with regard to the topic of communication, 2 points were awarded in cases where active and specific communication on the topic by healthcare professionals and communication experts was reported; 1 point in cases where active but non-specific communication was reported; 0 points when there was no active communication. On the topic of organizational procedures, 2 points were awarded when an active pathway was reported according to the criteria of maximum effectiveness, efficiency, and patient-centeredness; 1 point when an active but potentially improvable pathway was reported; 0 points for no active pathway.

An additional specific assessment was conducted, focused on the actual content of the web pages available on the websites of each of the 9 LHUs regarding the topic of vaccinations for adults outside the call-by cohort. The most important parameters to consider on such web pages have been identified by Italian [21,22,23] and international [24] technical groups in the field of digital communication in healthcare. Based on the recommendations provided by said technical references, a score from 1 to 5 was awarded for each parameter. The parameters and scores are combined in the matrix shown in Table S1.

### 2.4. Defining Strategies to Close the Gap between Ideal Procedure and Real Practice

The regional group responsible for the procedure “HPV vaccination for women treated for high-grade HPV-related cervical lesions” (November 2020) and for the present study analyzed the completed questionnaires and examined the LHU websites. The 9 doctors participating in the survey were individually interviewed to analyze the causes of the gap between ideal procedure and real practice and identify shared solutions to tackle them. Then a collegial brain-storming meeting was conducted to discuss the feasibility and effectiveness of the possible solutions for critical issues. Finally, a checklist of strategies to support good practice was produced and shared for the experts to implement at their LHUs. These strategies were the outcome of an analysis of the practices in use at the different LHUs, the critical issues that emerged from the questionnaire and from the web pages, and the proposed solutions.

### 2.5. Quality Improvement Strategy Outcome Measures

In order to complete the analysis, data were extracted from the regional oncological screening database relating to women diagnosed with CIN2+ lesions before and after the adoption of the regional procedure regarding “HPV vaccination for women treated for high-grade HPV-related cervical lesions” (November 2020) [20], and cross-linked with data in the regional vaccination register to establish the vaccination status of these women and the time elapsing between a diagnosis of CIN2+ lesions and a first dose of HPV vaccination (time to the provision of care).

The data extracted were:

- (number of women diagnosed with CIN2+ lesions at cervical screening from 18 Nov 2020 to 30 June 2022 who received at least one dose of HPV vaccine after said diagnosis)/(total number of women diagnosed with CIN2+ lesions at cervical screening during the same period);
- (number of women diagnosed with CIN2+ lesions at cervical screening from 1 Jan 2019 to 17 Nov 2022 who received at least one dose of HPV vaccine after said diagnosis)/(total number of women diagnosed with CIN2+ lesions at a cervical screening during the same period).
- The resulting dataset was divided into four, based on the time to the provision of care (the interval between the diagnosis of CIN2+ lesions and a first dose of HPV vaccination): less than 3 months, 3–6 months, 6–12 months, and more than 12 months.

### 2.6. Statistical Analysis

Data extracted were represented as a distribution of both absolute and relative frequencies of a discrete quantitative variable. The median and 5th, 25th, 75th, and 95th percentiles of time to the provision of care were calculated.

Data deriving from the questionnaire were represented as a radar chart. The radar chart was depicted as a sequence of equiangular spokes, with each spoke representing one of the variables. The data length of a spoke is proportional to the magnitude of the variable for the data point relative to the maximum magnitude of the variable across all data points. A line is drawn connecting the data values for each spoke.

No ethical approval was required, and data was communicated by Local Health Units in aggregated form.

**Figure 1:**
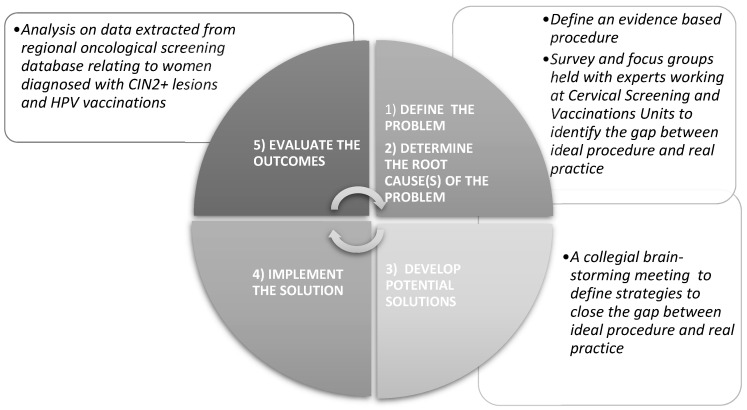
Conceptual framework of the Continuous Quality Improvement (CQI) project carried out by the Veneto Region Prevention Directorate regarding the HPV vaccination in women diagnosed with CIN2+.

A framework of the QI strategy adopted by the Veneto Region to analyze and improve the healthcare provider regarding HPV immunization for women with CIN2+ lesions is represented in Figure 1.

## 3. Results

**Figure 2:**
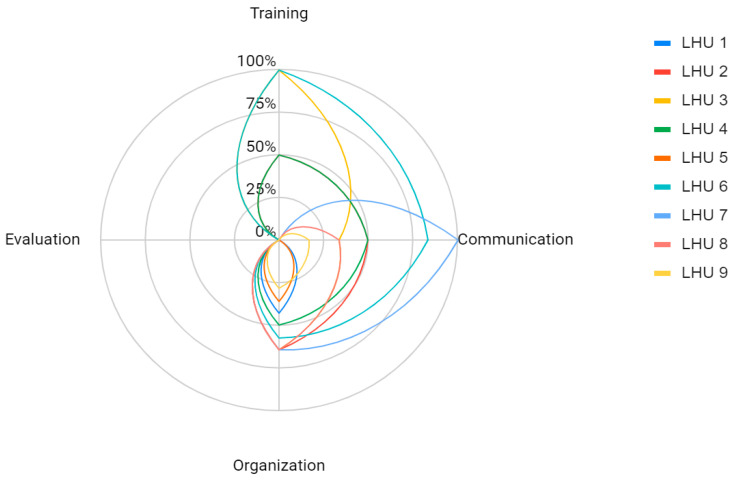
Overall results of the survey by Local Health Unit (LHU).

Figure 2 shows the data obtained on the gap between ideal procedure and real practice regarding the provision of HPV vaccination for women diagnosed with CIN2+ lesions at routine cervical screening. The nine LHUs differed considerably in each step of the pathway considered.

Table 2 shows the findings of the analysis conducted on the LHUs’ web pages relating to HPV vaccination for women diagnosed with CIN2+ lesions. Here again, the LHUs’ web communications on the topic varied greatly. Some LHUs have a dedicated web page explaining the details of the regional procedure. Others provide only general information about HPV vaccination and/or the papillomavirus, with nothing about the benefits of vaccination for women with CIN2+ lesions. One LHU has a web page that only provides contact details of vaccination clinics but no information about what vaccinations are available.

Table 3 shows the strategies adopted to improve the quality of healthcare provision in this setting. The most interesting cases concerned:

- strategies to improve the training of healthcare personnel: at two LHUs, they involved webinars or face-to-face meetings held by an expert (the doctor in charge of 2nd-level screening or an oncologist); the other LHUs engaged in active training and in-house meetings (of gynecologists) to discuss the procedure; the various LHUs also arranged for the proper storage and availability of printed copies of the procedure for easy consultation;
- strategies to develop effective communications: explanatory videos and video interviews were prepared and broadcast via social channels in two cases, and television interviews were used in another (and the material was edited by both healthcare specialists and communication experts); in one case, printed information sheets were produced and distributed at most of the LHUs’ vaccination clinics;
- strategies to develop an organized and proactive patient involvement: immunization was recommended in letters notifying patients that their cervical screening had revealed CIN2+ lesions; one LHU established a routine procedure for booking a vaccination within 30 days after surgery for CIN2+ lesions and, if the patient failed to attend, vaccination was recommended again at the time of her gynecological follow-up.

Table 4, Table 5 and Table 6 show data from the regional records, comparing women diagnosed with CIN2+ at 1st-level cervical screening and then vaccinated against HPV before and after the quality improvement efforts.

After the quality improvement strategies were implemented, one in every two women (as opposed to 30.85% beforehand) was given a first dose of HPV vaccine within 3 months of being diagnosed with CIN2+ lesions at 1st-level cervical screening, and the median time to the provision of care dropped from 158 to 90 days.

## 4. Discussion

The present analysis revealed an initially heterogeneous application by LHUs in Veneto of the regional procedure regarding the offer of HPV vaccination to women treated for CIN2+ cervical lesions. A quality improvement program produced a marked reduction in the time elapsing between a diagnosis of CIN2+ lesions on cervical screening and the administration of a first dose of the HPV vaccine.

The present study was conducted by the Prevention Directorate of the Veneto Region to improve the quality of the HPV vaccine provision to the specific at-risk population of women who have not undergone vaccination and can be compared with other experiences in Italy and Europe. For example, in Catalonia, Del Pino, and colleagues [8] analyzed the variation in adherence to HPV vaccination after cervical surgery, before and after the updating of regional supply policies, and found that vaccination adherence had more than doubled after the decision to offer the vaccine free of charge to this specific population. Moreover, in Taranto, Apulia Region, Italy, the local health authority has defined a new clinical pathway whereby gynecologists recommend treated patients get vaccinated, ideally within ten days after surgery [25]; furthermore, in Tuscany and in Rome, women with CIN2+ lesions are now vaccinated against HPV on the same day as cervical surgery, and receive the second dose on the day of the first gynecological follow-up after surgery [26,27].

The current analysis can be compared with the picture emerging from a recent review by Pereira et al. [28], who analyzed the main strategies used to implement clinical practice guidelines. The Authors described a variety of possible ways to implement healthcare guidelines, ranging from care pathways, education, and community action to health workers’ education and training, audits, or patient-directed interventions. The most often reported interventions involved the distribution of educational materials, meetings, reminders, audits, and feedback. Action on organizational culture, educational interventions, and reminders proved effective in promoting physicians’ adherence to the guidelines.

Our findings suggest that—for the training of general practitioners and other physicians involved in the pathway investigated here (primarily gynecologists, but also hygienists, oncologists, and other health professionals involved in screening or vaccination services)—it is essential to conduct at least one active training event. This can be a meeting or a webinar, possibly with scheduled subsequent update sessions. This is because various studies showed that, even among healthcare professionals, there are often prejudiced attitudes or knowledge gaps regarding vaccines, or their importance is simply underestimated [29,30]. A recent literature review conducted by Pavlovic et al. [31] showed that the attitude of European health professionals towards the various vaccinations is a critical issue, underscoring the importance of training and active involvement of health professionals on the subject of vaccinations. Another US study conducted in 2014 [32] found that, even in the specific context of vaccinations for individuals with certain risk-raising conditions, there are sometimes large gaps between the recommendations in the guidelines and current clinical practice, partly due to medical staff lacking the necessary information.

As concerns communication aspects for health service users, the checklist drawn up in the light of the present analysis proposes the production of ad hoc information materials aimed at women diagnosed with CIN2+ cervical lesions in the present case. These materials should be prepared jointly by healthcare personnel with expertise on the topic (who decide the content) and personnel experts in communication (who decide how to present it). The information should be disseminated using all currently-available communication channels (social networks, websites, paper brochures) to reach as much of the population as possible. Considering the growing attention paid to the issue of vaccinations for individuals who are at higher risk due to their clinical and/or social conditions (an issue brought to the fore by the COVID-19 vaccination campaign), it is now high time to update healthcare service web pages. They should be designed to be straightforward and accessible, and the contents easy to maintain and revise. Digital and traditional formats should be used, complying with the recommendations and best communication practices. A plentiful body of literature confirms that targeted communication strategies increase awareness among service users and consequently help to increase adherence to the vaccination offer [33,34,35].

As for evaluating the service provided, none of the LHUs considered had yet devised indicators or corrective actions to improve their vaccination offer process. This is a work in progress for all Vaccination Services. Process indicators are key to monitoring the pathway and implementing any active calls or catch-up actions.

All these considerations are confirmed in the US standards and recommendations for adult vaccination [36,37]. Their recommendations are aimed at all healthcare professionals, not just those normally operating in the vaccination sector. They are based on the assumption that adults are often simply not aware that they have conditions warranting certain vaccinations, and the main determinant of their decision whether or not to get vaccinated is the advice of a health professional. Every time a patient accesses healthcare is the right time to assess their vaccination status. When this does not happen, an opportunity for preventive action has been missed. The CDC and National Vaccine Advisory Committee also emphasize the importance of monitoring vaccinations and making the data accessible to different health professionals so that their advice can be tailored to a given patient. This is clearly most important in the case of patients who are at greater risk due to a clinical or social condition.

## 5. Conclusions

In conclusion, although further efforts will be needed to consolidate good practices and offer solutions endorsed by a larger number of experts, this study confirmed the importance of standard operating procedures as part of efforts to provide more efficient and patient-centered healthcare. The present study focused on the offer of HPV vaccination to women revealing CIN2+ lesions on routine cervical screening, but most of the good practices and considerations that emerged could be equally applicable to other vaccinations aimed at other at-risk sub-populations.

Increasing the adherence of a specific target population to a recommended vaccination is not only proof of quality and efficacy for a healthcare system but also has a profound impact in terms of lives saved and cost-effectiveness. Indeed, according to an Italian health technology assessment on HPV vaccination in women treated for HPV-related precancerous lesions, adjuvant vaccination is cost-effective in general and especially from the second year after surgery and can reduce the risk of recurrence by up to 80% compared to no vaccination after surgery [9,25]. It is, therefore, reasonable to expect that a project that succeeds in increasing the number of women who get vaccinated within the first months after cervical surgery would be beneficial in maximizing the impact of this vaccination offer and ultimately in ensuring better survival and quality of life for women treated for CIN2+ lesions.

### Strengths and Limitations

This study has both strengths and limitations that need to be mentioned. The main strengths lie in its originality and the novel nature of the data: to our knowledge, it is the first study to have taken a quality improvement approach to preventive medicine and to have examined the timing of steps in an integrated pathway leading from cervical screening to HPV vaccination.

The main pitfall is that the data of time to the provision of care are not perfectly comparable between the two cohorts considered since 43% of women in the “after-operating-procedure” cohort had less than 364 days (or sometimes less than 180 days) passed since the CIN2+ diagnosis at the time of writing. For this reason, further evidence will be needed to consolidate our findings. Moreover, the present study has a predominantly methodological approach and did not analyze the specific immunization status of the minority of CIN2+ women who had already been vaccinated before (some might be vaccine failures, but also patients might not have completed the schedule or they might have been vaccinated with HPV2 or HPV4, which protected against a narrower range of malignant genotypes): this may be a hint for future research.

## Figures and Tables

**Table 1:** Pathway for offering HPV vaccination to women with CIN2+ lesions, developed in the light of focus groups held with experts working at Cervical Screening and Vaccinations Units.

Training		
Are there dedicated training courses for healthcare personnel involved in cervical screening and HPV vaccination pathways (recommendations, good practices, counseling techniques, co-administration, etc.)?		Questions 1, 2
How LHUs communicate with the population		
Social networks	Are there specific campaigns or messages promoting HPV vaccination?	Question 3
Website	Is there a dedicated web page? If so, does it provide basic information on vaccination, who to contact, and booking methods?	Question 4
Printed matter	Is there any printed information material available at the screening facility?	Question 5
Logistics and organization		
Definition of pathways, logistics, and personnel involved	Are there established pathways for offering and organizing vaccinations? Which staff are involved?	Questions 6, 7, 8, 9, 10, 14, 15, 16, 17, 18, 19, 20
Access modalities	How can the population access vaccination (active call, booking, gynecological counseling,…)?	Questions 10, 11, 12, 13
Evaluation		
Process	Are process indicators used in the implementation of the pathway?	Question 21
Quality	Have any quality improvement activities or interventions been carried out (internal audits, focus groups, etc.)?	Question 22

**Table 2:** Overall results of the web page analysis, by the Local Health Unit (LHU), according to the scores detailed in Table S1. The range of numbers is [1 = critical lack; 2 = lacking content; 3 = improvable content; 4 = suboptimal content; 5 = optimal].

	Dedicated Web Page	Web Page Accessibility	Text: Sentence Length	Text: Word Complexity	Text: Layout	Vaccination Booking Information
LHU 1	1	3	3	3	4	2
LHU 2	1	3	1	1	1	1
LHU 3	3	2	5	5	3	4
LHU 4	1	2	1	1	1	3
LHU 5	2	3	3	1	5	5
LHU 6	4	3	5	5	5	4
LHU 7	5	2	5	3	4	3
LHU 8	5	1	5	3	4	5
LHU 9	1	3	1	1	1	1

**Table 3:** Checklist of strategies to improve practices to reach the target population and implement the procedure effectively.

Training	
General practitioners, gynecologists	✓ At least one active training, e.g., webinars or face-to-face meetings with an expert on the subject (2nd-level screening responsible physician, oncologist, etc.), to be repeated whenever new guidelines are introduced, and at least every two years in any case.
How LHUs communicate with the population	
Social networks	✓ Posting of at least one video/tutorial/post per year on social networks, with the content edited by healthcare personnel and communication experts.
Website	✓ Dedicated web pages on LHU websites explaining which vaccinations are available to citizens, with specific reference to individuals with risk-raising conditions. ✓ Web pages meeting the requirements of national guidelines on digital communication in healthcare.
Printed matter	✓ Printed information sheets to be attached to letters inviting women for routine cervical screening or notifying the outcome of screening, and distributed in the clinics (with the content edited by healthcare personnel and communication experts).
Logistics and organization	
Definition of pathways, logistics, and personnel involved	✓ Systematic check on a woman’s HPV vaccination status at the time of 1st-level cervical screening. ✓ Systematic provision of information to all unvaccinated women on the importance of HPV vaccination at the time of 1st-level cervical screening. ✓ Written recommendation to book HPV vaccination for women who test positive for CIN2+ lesions at the time of 1st-level cervical screening. ✓ For women who test positive for CIN2+ lesions at the time of 1st-level cervical screening, systematic provision of written information about the opportunity for all women already vaccinated with HPV2 or HPV4 to be revaccinated with HPV9. ✓ Definition of a local pathway to guarantee optimal timing for booking a first dose of vaccine. ✓ Check whether a patient has been vaccinated during the gynecological follow-up and, if not, repetition of the written and oral recommendation to do so. ✓ Prompt registration of vaccinations at the time of administration. ✓ Managing informed consent: patients should be asked to sign a form that allows for data exchange between Screening Unit and Vaccination Unit to facilitate active calls and vaccination catch-ups.
Access modalities	✓ To systematically make vaccination accessible also to women who undergo conization surgery outside the LHU screening pathway, all gynecologists operating in hospitals and at territorial Units should receive instructions and contact details so that they can forward patients with CIN2+ lesions for vaccination, regardless of where they were screened or treated. ✓ Specific modalities (i.e., phone No. and/or e-mail address) should be in place for patients wishing to contact the service to: ∙ book or manage appointments; ∙ ask dedicated healthcare operators for clinical information.
Evaluation	
Process	✓ Managing informed consent: patients should be asked to sign a form that allows for data exchange between the Screening Unit and Vaccination Unit for purposes of monitoring, quality control, and process indicator development. ✓ Defining and periodically monitoring at least one process indicator: e.g., the time elapsing between conization surgery and the first dose of HPV vaccine, the vaccination uptake rate,...
Quality	✓ At least one activity a year to improve the quality of the provision of healthcare at the LHU level (internal audits, procedure reviews, focus groups,...).

**Table 4:** Distribution of women diagnosed with CIN2+ lesions at cervical screening, by HPV vaccination status, before and after the introduction of the regional procedure.

Adoption of Regional Procedure (Nov 2020)	Vaccinated only after a Diagnosis of CIN2+ Lesions [*n* (%)]	Already Vaccinated [*n* (%)]	Never Vaccinated [*n* (%)]	Total [*n* (%)]
Before (1 Jan 2019 to 17 Nov 2020)	1381 (46.97)	316 (10.75)	1243 (42.28)	2940 (100)
After (18 Nov 2020 to 30 June 2022)	1249 (50.65)	313 (12.69)	904 (36.66)	2466 (100)

**Table 5:** Distribution of previously-unvaccinated women by time elapsing between being diagnosed with CIN2+ lesions at 1st-level screening and a first dose of HPV vaccine, before and after the introduction of the regional procedure.

Adoption of Regional Procedure (Nov 2020)	Vaccinated only after a Diagnosis of CIN2+ Lesions [*n* (%)]	Up to 3 Months *n* (%)	3–6 Months *n* (%)	6–12 Months *n* (%)	More than 1 Year *n* (%)
Before (1 Jan 2019 to 17 Nov 2020)	1381 (100)	426 (30.85)	318 (23.03)	287 (20.78)	350 (25.34)
After (18 Nov 2020 to 30 June 2022)	1249 (100)	625 (50.04)	332 (26.58)	232 (18.57)	60 (4.80)

**Table 6:** Median time to the provision of care (i.e., days elapsing between a diagnosis of CIN2+ lesion at 1st-level screening and the administration of a first dose of HPV vaccine), and percentiles, before and after the introduction of the regional procedure.

Regional Procedure (Nov 2020)	Median (days)	5th Percentile (days)	25th Percentile (days)	75th Percentile (days)	95th Percentile (days)
Before (1 Jan 2019 to 17 Nov 2020)	158	23	78	367	857
After (18 Nov 2020 to 30 June 2022)	90	22	55	170	362

## Data Availability

Restrictions apply to the availability of these data. Data was obtained from Veneto regional records and are available from the authors with the permission of the Information Systems Unit of Azienda Zero.

## Supplementary Materials

The following supporting information can be downloaded at: https://www.mdpi.com/article/10.3390/vaccines11040757/s1, File S1: Questionnaire; Table S1: Matrix of the scores for each parameter explored in the analysis of the LHU web pages.
