# Supplementary material for: Improving the Quality of Healthcare Provision Regarding HPV Immunization for Women with CIN2+ Lesions: The Experience of the Veneto Region in Italy
Source: Vaccines (Basel). 2023 Mar 29;11(4):757. doi: 10.3390/vaccines11040757 (PMC10145840; doi:10.3390/vaccines11040757)
Supplement: Supplementary file 1 [file vaccines-11-00757-s001.zip › vaccines-2222708-supplementary.pdf]

vaccines

MDPI

File S1. Questionnaire

The 22-item questionnaire used to analyze Local Health Units' pathways leading from cervical screening to HPV vaccination in Veneto after the publication of a Regional Procedure (Nov 2020).

Name and surname: ______________________________

Qualification/role: ______________________________

Local Health Unit: ______________________________

1. Have general practitioners operating in the local area been actively informed about the offer of HPV vaccination for women treated surgically for CIN2+ lesions?

- □ Yes, they have. Please, specify how: ______________________
- □ No specific training on the subject has been actively offered to GPs.

2. Have gynecologists operating at the units affected by the Regional Procedure actively informed about the offer of HPV vaccination for women treated surgically for CIN2+ lesions?

- □ Yes, they have. Please, specify how: ______________________
- □ No specific training on the subject has been actively offered to gynecologists.

3. Have messages been launched via social networks to inform the population about the importance of vaccination against HPV, with particular reference to the offer aimed at women with CIN2+ lesions?

- □ Yes, messages (videos, photographs, posts, …) have been disseminated on the importance of HPV vaccination in general, and specifically on the offer of free HPV vaccination for women with CIN2+ lesions.
- □ Yes, messages (videos, photographs, posts, ...) have been disseminated relating to the importance of vaccination against HPV in general.
- □ Social channels have never yet been used to spread messages relating to this issue.

4. With regard to your LHU website, who took care of drafting the web page to inform people about the importance of vaccination against HPV, and how to access it, with particular reference to the offer aimed at women with CIN2+ lesions?

- □ Healthcare personnel (hygienists, resident doctors, healthcare assistants, ...) with the support of expert personnel (press office, communication staff, ...) for the communication aspects.
- □ Healthcare personnel (hygienists, resident doctors, healthcare assistants, ...).

- ◻ Personnel expert in communication.
- ◻ The LHU’s website does not yet have pages dedicated specifically to this type of vaccination offer.

5. In the rooms dedicated to cervical screening (clinics, waiting rooms, ...) is there information material (e.g. posters, flyers, ...) relating to the HPV vaccination offer for women found positive for CIN2+ lesions, and on the importance of such vaccination in general?

- ◻ Yes, there is generic information material about HPV vaccination.
- ◻ Yes, there is information material about the offer of HPV vaccination for women with CIN2+ lesions, with details on how to book the appointment.
- ◻ There are no posters or flyers on the subject.

6. At the time of cervical screening, how do healthcare staff at the clinic ask women about their HPV vaccination status?

- ◻ The healthcare staff usually inquire verbally about a patient's vaccination status.
- ◻ The healthcare staff usually check a patient's vaccination status in the vaccination database.
- ◻ The patient's vaccination status is not currently checked routinely by the healthcare staff at the time of cervical screening.

7. At the time of cervical screening or later, is any information/communication about the importance of HPV vaccination aimed at all women, even those who do not have CIN2+ lesions?

- ◻ Yes, with verbal recommendations from health professionals, generally after checking the woman's vaccination status.
- ◻ Yes, with a written recommendation in the letter communicating the outcome of the screening.
- ◻ Yes, with printed matter distributed at Screening, Gynecology or Vaccination Units (e.g. flyers, posters or other similar material).
- ◻ Yes, with both verbal and written recommendations (in the screening outcome letter or by other means).
- ◻ There is no specific and systematic communication/information on the topic for women who do not have CIN2+ lesions.

8. In the event of CIN2+ lesions being detected, what HPV vaccination promotion and counseling action is envisaged in the LHU's cervical screening pathway?

- ◻ An active invitation to an appointment via a letter from the Vaccination Service.
- ◻ A recommendation to get vaccinated in the letter communicating the finding of CIN2+ lesions on cervical screening, or similar;
- ◻ A recommendation to get vaccinated contained in the post-surgery letter written by the gynecologist, or in the information (attached to the regional Procedure or similar) provided at the Gynecology department;

- ◻ A recommendation to get vaccinated, usually given verbally by the gynecologist.

9. Has a systematic transfer of information between the Screening Unit and the Vaccination Service been organized at the LHU level?

- ◻ Yes, for purposes of tracking patients' acceptance of the offer;
- ◻ Yes, for monitoring and active call purposes;
- ◻ No, no such information transfer is currently active;
- ◻ Not relevant (the vaccination is administered at the Gynecology Unit).

10. Are pathways defined for the active offer of HPV vaccination also to women who have PAP tests or HPV tests elsewhere, not at the LHU's screening unit (e.g. with a note in the anatomopathological report, ...)?

- ◻ Yes, all women diagnosed with CIN2+ lesions are actively offered vaccination, regardless of where they go for testing. Please, specify how:

____

____

- ◻ Yes, all women who have these tests are actively offered vaccination in the event of a CIN2+ lesion being detected. In the case of a negative test outcome or lower-grade lesions, women are only informed about the importance of vaccination. Please specify how:

____

____

- ◻ No, there are currently no established methods for actively offering vaccination to women with CIN2+ lesions diagnosed elsewhere.

11. In the case of patients undergoing surgery for CIN2+ lesions who had already been vaccinated with HPV2 or HPV4, is revaccination with HPV9 recommended?

- ◻ Yes, systematically.
- ◻ Yes, though not systematically.
- ◻ No, or only seldom.

12. Once patients with CIN2+ lesions have been informed of the importance of vaccination, how is the vaccination appointment booked?

- ◻ By active call with a letter delivered to their home.
- ◻ Directly at the time of patients accessing the Gynecology unit for conization;
- ◻ By patients themselves, using a dedicated telephone line (or other channel) to book vaccinations that also affords them the opportunity to speak with a healthcare professional.

- ☐ By patients themselves, by calling the Vaccination Service or Gynecology Unit, but not on a dedicated telephone line or other channel (i.e. through a call center with administrative staff).

13. Is there a dedicated telephone or email contact specifically for the HPV vaccination of women treated for cervical lesions, to deal with patients' unexpected needs (e.g. for further explanations, booking or rescheduling the appointment, ...)?

- ☐ Yes, there is a dedicated telephone line for booking HPV vaccination for women with CIN2+ lesions (and possibly other categories entitled to vaccination free of charge outside the cohort call). Please indicate the time slots and days of the week when this line is available to potential users:

____

____

- ☐ Yes, there is a mailbox and a dedicated telephone line for booking HPV vaccination for women with CIN2+ lesions (and possibly other categories entitled to vaccination free of charge outside the cohort call). Please indicate the time slots and days of the week when this line is available to potential users:

____

____

- ☐ No, the contact is the LHU's general call center and email address for vaccinations.

14. Where is the vaccination administered?

- ☐ At the Gynecology Unit.
- ☐ At the Primary Care Department's specialist gynecology clinic.
- ☐ At the Prevention Department's Vaccination Service.
- ☐ At the Gynecology Unit, at the Prevention Department, and at any other access points. In this case, please specify how many, and which access points are used for HPV vaccination at the LHU level:

____

____

- ☐ Other. Please specify: ______________________________

15. Which healthcare professional is primarily responsible for administering HPV vaccination to women with CIN2+ lesions?

- ☐ Healthcare assistant
- ☐ Nurse
- ☐ Hygienist
- ☐ Gynecologist

16. If the vaccination activity is also managed by personnel outside the Vaccination Service, how are the vaccinations administered and recorded in the regional portal?

- □ Directly by the operator at the time of administration.
- □ Later, but systematically by the healthcare operator administering the vaccine.
- □ Informally and later transmitted to the Vaccination Service staff for recording in the regional portal.
- □ No recording modality has been defined as yet.
- □ Not relevant (vaccinations are administered only by the Vaccination Service).

17. Are there catch-up actions for women treated surgically for cervical lesions who do not adhere to the active offer of HPV vaccination?

- □ Yes, with the periodic retrieval of lists and telephone contacts, with the possible registration of dissent or booking of a new appointment.
- □ Yes, other. Please, specify:

____

____

- □ No, there are currently no catch-up actions for women who do not adhere to the offer.
- □ No, there are currently no catch-up actions for women who do not adhere to the offer due to obstacles related to the protection of patient privacy in data transmission.

18. At your LHU, HPV vaccination is administered free of charge to women with CIN2+ lesions:

- □ Only for up to one year after conization.
- □ Often without any time limit.
- □ Without any time limit.

19. Have there been specific critical issues in the implementation of the regional procedure? If so, please specify.

____

____

20. Since when (indicate month and year) has the active offer of HPV vaccination pathway for women undergoing surgery for CIN2+ lesions (established by the Regional Procedure issued in Nov 2020) been active at your LHU?

____

21. Have process indicators been identified at your LHU (e.g. percentage of adherence, time elapsed between surgery and vaccination, or other indicators) regarding the implementation of the process envisaged by the Regional Procedure issued in November 2020? If so, are the indicators used for structured periodic monitoring?

□ Yes. Please describe the indicator: ________________________________

□ Not yet.

22. Have Quality Improvement initiatives been undertaken yet at your LHU regarding the active offer of HPV vaccination described in the Regional Procedure issued in November 2020 (internal audits, focus groups, ...)?

□ Yes. Please describe the indicator: ________________________________

□ Not yet.

Table S1. Matrix of the scores for each parameter explored in the analysis of the LHU web pages.

SCORE	1	2	3	4	5
**Dedicated web pages**	No page dedicated to vaccinations for women of childbearing age and/or subjects with risk-raising conditions, and/or containing further information, apart from contact details of vaccination services with minimal information about the vaccinations available.	Information page on immunization in general (with contact details of vaccination services with minimal information about the vaccinations available), plus additional material on vaccinations offered for women of childbearing age and/or HPV vaccination, and/or vaccinations for people with risk-raising conditions, contained in attachments or on other pages not immediately available to the user.	A Page dedicated to vaccinations for women of childbearing age and/or HPV vaccination, without any specific information about the offer of vaccinations free of charge for individuals with risk-raising conditions.	A Page dedicated to vaccinations for women of childbearing age and/or HPV vaccination, mentioning the offer of vaccinations free of charge for individuals with risk-raising conditions, but without providing specific details.	A Page dedicated to vaccinations for women of childbearing age and/or HPV vaccination, with specific details about the offer of vaccinations free of charge for individuals with risk-raising conditions.
**Web page accessibility**	Difficult to access: 5 or more clicks are needed and/or titles are not user-friendly, or the page cannot be found by entering keywords in the search bar on the home page.	Moderately complex: 2-4 clicks on titles that can be found only with careful reading and are not always very user-friendly, or users need to carefully scroll down to the suggested pages when users enter keywords in the home page search bar.	Limited complexity: 2-4 clicks on titles that can be found quickly and are user-friendly, or among the first pages suggested when users enter keywords in the search bar on the home page.	Easy: a single click on a readily-identifiable title on the home page.	Very easy: just one click on a highlighted title on the home page.
**Sentence length**	Text is difficult to understand due to frequent use presence of long sentences characterized by numerous (4 or more) coordinate or subordinate clauses (or no text.)	Text is sometimes difficult to understand due to some long sentences (sometimes with 3 or more coordinate or subordinate clauses).	Text containing by generally-acceptable short sentences, with up to 3 subordinate or coordinate clauses (mostly 1-2).	Text is characterized mainly by short periods, with no more than 2 subordinate or coordinate clauses.	Text is characterized by short periods, with no more than 2 subordinate or coordinate clauses (mostly 1).
**Word complexity**	Repeated and systematic use (1 or more per sentence) of technical terms, Anglicisms, Latinisms, unexplained abbreviations and acronyms (or no text.)	Repeated presence (> 1 per sentence) of technical terms, Anglicisms, Latinisms, unexplained abbreviations and acronyms.	Sporadic presence (1 or more in proportion to the length of the text) of technical terms, Anglicisms, Latinisms, unexplained abbreviations and acronyms.	Sporadic presence (1 or more in proportion to the length of the text) of technical terms, Anglicisms, Latinisms, abbreviations and acronyms that could be avoided, but that are always explained.	No technical terms, Anglicisms, Latinisms, abbreviations or acronyms, or - when necessary – they are always explained.
**Layout**	Titles and subtitles are lacking or difficult to distinguish from the body of the text. Blank spaces are lacking or inadequate (or no text.)	Poor or unclear titles and subtitles; limited blank spaces, with generally dense text.	Clear titles and subtitles; limited blank spaces, with a tendency of the text to be dense.	Fully adequate titles, subtitles and blank spaces in the text to make it easier to find information within the text.	Titles, subtitles and blank spaces in the text fully adequate to simplify the retrieval of information within the text, with the aid of drop-down menus, buttons to expand the text, or other similar IT tools to facilitate reading.
**Vaccination booking information**	On the website there is only a home page containing the contact details of the vaccination	Booking modalities and contact details are not available on the home page (and there are no	Booking modalities and contact details are not available on the home page, but are available in	Booking modalities and contact details are not available on the home page, but there are easily	Booking modalities and contact details clearly indicated on the home page.

	clinics.	links to the booking page (a second search on the website is necessary).	the form of an attachment or link that is not immediately available on the homepage.	accessible links to the booking page. Or they are available on the home page with limited prominence or sparse detail.	
